# History and Development of TCM Case Report in a Real-World Setting

**DOI:** 10.1155/2021/7402979

**Published:** 2021-12-29

**Authors:** Hua Zeng, Yiqi Qiao, Xue Luo, Xin Chen, Zhendong Wang, Huafeng Pan, Qi Wang, Guo-qing Zheng

**Affiliations:** ^1^Science and Technology Innovation Center, Guangzhou University of Chinese Medicine, No. 12, Jichang Road, Baiyun District, Guangzhou 510405, China; ^2^Department of Neurology, The First Affiliated Hospital of Zhejiang Chinese Medical University (Zhejiang Provincial Hospital of Chinese Medicine), No. 54, Youdian Road, Shangcheng District, Hangzhou 310006, China

## Abstract

**Objective:**

The medical record of Chinese medicine is a miniature of the theoretical system of traditional Chinese medicine (TCM), with a time-honored history in a real-world setting and a firm place in medicine. In modern times, people have emphasized the value and standardization of TCM cases. The aim of this study was to explore the historical origins and developments of TCM case records.

**Methods:**

A chronological narrative style was used to divide the development history of TCM case records into early (1600 BC–220 AD), middle (220–1911 AD), and modern periods (1912–till now). The historical context of the origin and development of TCM case records was analyzed through the evolution of the format and content of the case recording files with the specific documents and distinctive cases.

**Results:**

From the early to middle period, the development of TCM case record had experienced four periods: the budding, blossoming, maturity, and heyday. In modern times, they presented the following characteristics: A, the establishment and development of the discipline of TCM medical records; B, the standardization of the writing format of TCM medical records; C, a large number of books concentrating on recording and studying TCM medical records, especially those of prestigious veteran TCM doctors; D, the proliferation of TCM case reports published in journals; E, the establishment of TCM medical records databases and application platforms integrating computer programs and artificial intelligence; F, many reporting guidelines have been developed in order to improve the reporting quality of case report in TCM.

**Conclusions:**

The study analyzed and illustrated the characteristics of TCM case records of different dynasties in terms of writing content and format. TCM case record is a relatively young discipline in spite of its ancient origins. TCM case records still have far-reaching significance for the inheritance and development of TCM theory and clinical experience. From the wisdom of history, its positive impact has just been revalued to be validated and it will continue to develop.

## 1. Introduction

On the issue of terminology in this study, “case record” is used extensively in various aspects, whereas “case report” is used exclusively in modern medical journals. Case report refers to a detailed description and formal summary of a diagnostic or therapeutic problem experienced by one or several patient(s), such as exposure, symptoms, signs, interventions, and outcomes [1, 2]. The early case records in the west can be found in the ancient Egyptian medicine papyrus records from about 1600 BC [3–5]. In modern times, the case report is a particular form of clinical evidence despite the low-ranking evidence level in the modern evidence hierarchy [6]. It plays important roles in identifying new, rare, or unusual diseases [7]; evaluating the efficacy, safety, and cost of treatments; and improving medical education, patient care, and medical research. Vandenbroucke [8] stated that “case reports and series are highly sensitive to the discovery of novelty and therefore remain one of the cornerstones of medical progress, providing many new perspectives in medicine.”

Traditional Chinese Medicine (TCM) is a unique and integrated medical theoretical system with a long history of several thousand years. Its origin can be traced back to remote antiquity. Historically, case records are a delicate and irreplaceable form of reporting on the comprehensive application of TCM theories [9], a great treasure of TCM heritage, and a product of its long-term development. The possibly earliest TCM case records date back to the Yin and Shang Dynasties (1300-1046 BC). When hieroglyphics were invented, medical records were engraved on cattle bones and tortoise shells [10, 11]. Case records/case reports have been providing evidence for effectiveness and safety of TCM in a real-world setting, which has been accumulated over thousands of years. Its connotations are the following: (1) It originates from clinical practice with integrity and authenticity; (2) it is hands-on, immersive case of treatment; (3) it describes individual syndrome differentiation and treatment, which is the personalized design and application of the TCM theory; and (4) it is clinically instructive and exemplary for future generations [12, 13].

The wisdom of the long history of TCM has blossomed by taking history as an inspiration to guide the future development. For example, the inspiration for artemisinin for malaria came from Ge Hong's (284–346 AD) “A handful of Artemisia annua, soak in two liters of water, wring out the juice and drink it all” from the *Zhouhou Beiji Fang* (*Handbook of Prescriptions for Emergencies*) [14]. *Report to ministers from the Department of Health Steering Group on the Statutory Regulation of Practitioners of Acupuncture*, *Herbal Medicine*, *Traditional Chinese Medicine and Other Traditional Medicine Systems Practised in the UK* [15] pointed out that “the recorded history of traditional use over many years should be assessed and incorporated into the evidence base supporting the effectiveness and safety of herbal/traditional medicines and acupuncture” when building an evidence base. The *European Directive on Traditional Herbal Medicinal Products* [16] also noted that “The long tradition of the medicinal product makes it possible to reduce the need for clinical trials insofar as the efficacy of the medicinal product is plausible on the basis of long-standing use and experience” (Directive 2004/24/EC). Thus, it is necessary to explore chronologically the historical origin and development of TCM case records and their contribution to the modern innovations and developments of TCM.

## 2. Early Period

The early period of TCM case records was from Shang Dynasty (1600-1046 BC) to Han Dynasty (206 BC–220 AD). The summary of their main contributions in the period is shown in Table 1.

**Figure 1:**
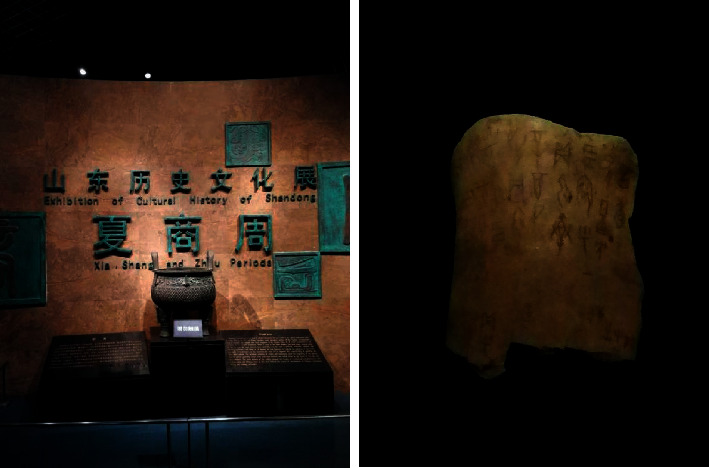
The oracle bones of buffalo and tortoise shells in Shang Dynasty, which we obtained from the Shandong Museum in Shandong province, China.

During the Shang Dynasty, medical principles were based on myths and legends as well as experience, which were very primitive in form. Inscriptions emerged on oracle bones of buffalo and tortoise shells (Figure 1), some of which described the use of wine and hot water as medicine and stone needles and bronze knives as surgical instruments. Some oracle bones threw light on illnesses, which were divination records about symptoms and signs, disease patterns, etiology, diagnosis, dynamic course of disease development, and interventions. It seemed as the earliest records about medical cases and the embryonic form of TCM case records [17].

Zhou Dynasty (1100-221 BC) was divided into four periods: Western Zhou (1100-771 BC), Eastern Zhou (700-256 BC), Spring and Autumn Period (770-476 BC), and Warring States Period (476-221 BC). TCM achieved in an extent of development during Zhou Dynasty especially in medical organization. The *Zhouli* (*Rites of Zhou*) [18, 19], written by Zhou Gong-dan, is an important document that records the ceremonies and systems from Western Zhou Dynasty to Warring States Period. He played a valid role in the consolidation and development of Zhou Dynasty and in the establishment of national laws and regulations. The book consists of six parts corresponding to six ministries according to ancient cosmology. One part named the celestial ministry, recorded that there was an organized medical system in the Eastern Zhou Dynasty in which the imperial officials received various medical training. TCM doctors were classified into four categories: *shiyi* (dietitian), *jiyi* (physician), *yangyi* (surgeon), and *shouyi* (veterinarian) [20]. Their duties were well defined. The chief doctor was responsible for the administration of all medical matters and the collection of drugs for medical purposes. The doctors' works were examined based on their case records recorded by the chief doctor [21]. Each doctor's salary was fixed according to the case recording results shown at the end of the year. If all cases get well, it is excellent; if there is one failure in ten cases, it is second; if two out of ten, third; three out of ten, fourth; and if four out of ten, it is bad. When any death occurs, the doctor in charge has to record the cause of death and submit the report to the superintendent.

During the early Spring and Autumn Period, *Lvshi Chunqiu* (*The Spring and Autumn of Lv Bu-wei*) [22], an encyclopedic Chinese classic document compiled around 239 BC, had recorded the budding of TCM case record. It recorded the following case of psychotherapy:

  Case 1: King of the Qi state (323-284 BC) suffered from *Wei* (sore and ulcer) during the Warring States Period. Wen Zhi, a proficient TCM physician at the Song state, was invited to treat him. After detailed diagnosis, he said to the prince: “Your father is treatable, but he will kill me after cured.” The prince asked: “Why?” Dr. Wen said: “The only treatment method is to infuriate your father. If I do it, I am surely put to execution.” However, the prince begged: “I pledge my life and my mother's life for you.” Dr. Wen had appointments with the prince to cure the King, but he deliberately broke them numerous times. Consequently, he incurred the wrath of the King. Then, Dr. Wen kept the appointment with King to ignite his fury through the following behaviors: he went to King's bed without taking off his shoes and deliberately trampled on King's clothes. Subsequently, Dr. Wen inquired the medical details; however, the King refused to reply. Dr. Wen further intentionally attacked the King with rude language, causing that the King raised the devil. He was so angry that he shouted loudly and sat up. These treatments cured the King's disease.
  Comments: It is similar to psychotherapy in modern medicine. Although the case was not recorded by the doctor himself, it already contains three important parts of the case record: the schedule, diagnosis, and treatment of the disease. However, it lacks patient's information and doctor's opinion in detail.

The Han Dynasty (206 BC-220 AD) was a time of innovation with great developments in arts, philosophy, and technology. The ancient Silk Road in Northwest China, a popular communication and trade route, played key roles in promoting the development of TCM. The buds of TCM case records burst into a gorgeous blossom in the Dynasty. In addition, books on the study of ancient Chinese history appeared. For example, the *Shi Ji* (*The Historical Records*) [23], completed by Sima Qian (145-85 BC), was described in detail of China's history from the earliest times to his own days. There were 25 TCM cases recorded in the biographical section of *The Historical Records*, named *Bian Que & Cang Gong Liezhuan (Biography of Bian Que and Cang Gong)*, which was probably the earliest extant and relatively complete TCM case records in Chinese historical literature [24]. *Cang Gong*, also named Chunyu Yi (215-167 BC), was the only physician going down in history in the Western Han Dynasty [25]. He was the first to record clinical cases through personal observations with the motivation to evaluate the percentage of successes and failures and find a guide for future predictions. He said: “The record of medical cases should be a conscious behavior of TCM physicians to summarize the success or failure and to improve the medical level.” The 25 cases of him reflected the change of thinking from passive assessment to active case compilation in the Western Zhou Dynasty. The main characteristics and academic thinking of these cases are as follows: A, it pioneered the writing content and format of TCM case record, which included patient' name, home address, profession, name of disease and name of TCM pattern, main complaint, pulse condition, etiology, pathogenesis, syndrome differentiation, diagnosis, treatment, and prognosis; B, 23 kinds of diseases were recorded, most of them were digestive diseases. Failures as well as successes were noted as they really happened. Fifteen out of 25 patients were successfully cured; C, he ascribed most illnesses to excess sweating or excess sexual life or over-drinking or over-fatigue or exogenous pathogenic factors such as wind or cold; D, he paid much attention to the diagnosis of observation and pulse-taking; E, he was skilled at flexibly using acupuncture, moxibustion, Chinese herbal medicine, cold compress, and other treatment methods. Chinese herbal medicine was used in the form of decoction, powder, medicated liquor, gargle, and even pill. He is thought to be the first person to use pills as a form of drugs, such as pill of Banxia (*Rhizoma Pinelliae*). The following is a case record of his style:

  Case 2: An officer in the Qi state suffered from dental caries. Dr. Chunyu asked him: “Do you rinse your mouth after eating?” The officer said: “No, I do not.” Dr. Chunyu asked again: “Will you open your mouth when sleeping?” He said: “Yes, I always do that.” “And do you have any other discomfort,” Dr. Chunyu inquired. The officer answered: “I have got a wind-cold these days.” Dr. Chunyu said: “Ok, I will do moxibustion with your left Yangming meridian, and give you a prescription named Kusen (*Radix Sophora Flavescentis*) decoction, you must gargle it 600 ml a day.” The official was confused and asked: “Dear doctor, why do I have cavities?” Dr. Chunyu answered: “I think the causes included that you had attacked with evil-wind, slept with mouth opened, and did not rinse the mouth after eating.” Then, the officer did moxibustion every day and rinsed his mouth with Kusen decoction (post-cibum). Several days later, he recovered.
  Comments: This case record is more mature in writing style. It is closer to modern guidelines of case record as it contains the patient information, diagnosis, treatment interventions, and the clinical efficacy. It is scientific and advanced at that time to gargle with *Radix Sophora Flavescentis* decoction, which was proved to have a variety of pharmacological activities, such as anti-inflammatory and antiviral in modern [26].

## 3. Middle Period

The middle period was defined from the Three Kingdoms (220–280 AD) to the Qing Dynasty (960–1911 AD). The development of TCM case records had achieved unprecedented development. There had a huge change took place at the Song Dynasty (960–1279 AD). With the progress of publishing and printing technology and the attention to medicine paid by the Song' government, medicine practice was becoming more reinforced and specialized. Monographs on specialized fields or particular diseases were appeared. At that time, the publications' number and variety transcended the sum of all previous dynasties, many of which were quite original. The summary of main contributions in the period is shown in Table 2.

Xu Shu-wei (1080–1154 AD) was not only an expert on exogenous febrile disease, but also having a greatly influence on the treatment of miscellaneous diseases. One of his great contributions *Shanghan Jiushi Lun* (*Ninety Syndromes of Typhoid Diseases*) [27], written in 1133 AD, was the first existing TCM case records monograph, and the earliest collection of typhoid case records. The book described ninety cases of typhoid, most of which recorded the following aspects: A, patient information: (a) demographic information such as appellation, age, gender, address, occupation, diet, and lifestyle; (b) main symptoms of the patient (his or her chief complaints, tongue, and pulse); B, time line: important dates and times in this case, including time of onset and duration, and time of consultation, as well as the 24 solar terms if related to the case); C, diagnostic reasoning; D, the utilization of herbal formulas and treatment efficacy. The effectiveness was 78 treatable cases and 12 incurable cases. He used 78 prescriptions in total, and 42 out of them were classical TCM prescriptions. His second great contribution was *Puji Benshi Fang* (*Prescriptions of Formularies for Universal Relief*) [28] (1143–1154 AD), the representative formularies that contained a total of 127 cases. It included 113 cases of miscellaneous diseases treated by himself and 14 cases by his peers. The TCM case records were compiled in this book in the form of an appendix. He was the pioneer to record clinical cases behind his prescriptions in order to expound TCM theory, which had an impact on the format of subsequent TCM case record.

*Xiao'er Yaozheng Zhijue* (*The Appropriate Way of Recognizing and Treating Infant Maladies*) [29] was the earliest extant, well preserved, and complete monograph of paediatric case records in China written by a disciple of Dr. Qian Yi. Qian Yi (1035–1117 AD), an outstanding paediatrician in the Northern Song, was known as the father of TCM paediatrics. This book has a total of three volumes, and the middle volume contains Dr. Qian's 23 case records. He recorded different various kinds of diseases such as fever, vomiting and diarrhea, cough and asthma, skin rash, acute or slow convulsion, hemoptysis, dyspepsia, and ascariasis. He was the pioneer to differentiate the symptoms of diseases. When coming down to treatment, Dr. Qian emphasized on the relationship between the onset time of disease and the season.

During the long-term development of the TCM theory, due to the diversity of geography, time, and academics, a medical phenomenon with very distinctive characteristics has emerged, namely, the formation of different academic schools of TCM. Its birth has far-reaching significance in promoting the inheritance and development of TCM theories and TCM case records. Academic schools can be formed actively or passively, and they require specific “soil conditions,” that is, development background, such as economic development, cultural prosperity, medical and commercial gathering, and academic exchanges. The main characteristics of TCM academic schools are a unique academic idea or proposition, a specific style of medicine, or a certain treatment technique or characteristic therapy as a heritage, and a self-contained system with a certain historical influence and recognition. TCM academic schools must have typical representatives and representative works, and a stable inheritance system. Before the Tang and Song dynasties, most of the medical practitioners of all generations referred to the classical Chinese medical works such as *Huangdi's Internal Classic* (*Huang Di Nei Jing*), *Difficult Classic* (*Nan Jing*), *Treatise on Cold Damage Diseases* (*Shang Han Lun*), and *Synopsis of the Golden Chamber* (*Jin Gui Yao Lue*), so they could be passively divided into the School of Medical Scriptures, the School of Scriptures, and the School of Typhoid. However, due to their lack of clear and continuous mentor-apprentice system (“Shicheng” system), some scholars believed that the classical classics should not be considered academic schools. It was not until the Jin-Yuan period, when the four great scholars of Liu Wansu, Zhang Congzheng, Li Dongyuan, and Zhu Danxi each formed a different academic doctrine and a relatively independent academic school that TCM academic schools began to be classified according to strict requirements [30].

In the Jin Dynasty, Liu Wansu started to study the *Plain Questions* (*Su Wen*) at the age of 25. He took the “nineteen articles of pathogenesis” of *Plain Questions* as the theoretical basis, systematically classified diseases according to the five evolution phases and six climatic factors (Five Yun and Six Qi), analyzed the etiology of diseases, and combined his clinical experience and academic thought to explore the inner connection between causes and symptoms, and put forward his own insights in diagnosis and treatment methods. This led to the creation of the book *Plain Questions on Concept of Original Disease Type* (*Su Wen Bing Ji Yuan Bing Shi*), in which he proposed that “six climatic factors can transform fire” and founded the He-Jian School, also known as the Fire-Heat School. He also proposed the principles of cooling the exterior of the body and dipping heat to nourish yin as the treatment of heat-related diseases, which laid the foundation for the formation of the later Warming Diseases School. Zhang Congzheng, a follower of Liu Wansu, founded the Eliminating Pathogenic Factor School, emphasizing the academic idea of “disease is born from pathogenic factors,” and developed the method of eliminating pathogenic factors by sweating, vomiting, and coming down methods, which also influenced the Warming Diseases School. He also authored the signature work *Confucians' Duties to Parents* (*Ru Men Shi Qin*). According to Li Dongyuan of the Jin Dynasty, “internal injury to the spleen and stomach leads to all kinds of diseases.” He believed that “Inadequate spleen and stomach lead to many diseases.” Based on the theory of Huangdi's Internal Classic, he thought that the four seasons are all based on nourishing stomach qi, so his treatment emphasized on regulating the spleen and stomach and raising the middle qi, and he made new formulas such as Buzhong Yiqi decoction. Because he was good at regulating the spleen and stomach with warm tonic methods, later generations called the academic school represented by him the Tonifying Spleen School. The representative work of the Tonifying Spleen School is *Treatise on the Spleen and Stomach* (*Piwei Lun*). In the Yuan Dynasty, Zhu Danxi was the founder of the Nourishing Yin School. He was the third-generation disciple (direct student) of Liu Wansu, the founder of the He-Jian School. He studied the doctrines of the above three schools and created the theory that “Yang is always in excess and Yin is always in deficiency,” advocating the nourishment of Yin. He advocated the doctrine of nourishing yin with Yin deficiency and Yang hyperactivity as the core and created the famous formula, Yue Ju Pill. The representative work of the Nourishing Yin School is the *Danxi's Mastery of Medicine* (*Danxi Xinfa*), which was compiled by his disciples and focuses on miscellaneous internal diseases and the Danxi doctrine, discussing in detail the disease name, cause, syndrome, identification, symptoms, and treatment prescriptions. The Nourishing Yin School had an important influence on the formation and development of the later Xin'an school of medicine. In summary, the main characteristics of TCM academic schools in Jin and Yuan Dynasties are shown in Table 3.

During the Ming Dynasty (1368–1644 AD) and Qing Dynasty (1616–1912 AD), the development of TCM case record entered a relatively stable stage. With the increase of records of TCM cases in literature in previous dynasties, the number and variety of TCM case records have increased, and the volume of TCM case record has formed a certain scale, which laid a literature foundation for doctors in Ming and Qing Dynasties to summarize the medical cases of predecessors. The large amount and the standard style of TCM case records have become a symbol of the development of TCM case record towards prosperity. Ming Dynasty is the mature period, and Qing Dynasty was the heyday of the development of TCM case record. The main characteristics in this period were as follows: A, many experts begin to work on the standardization of medical case writing, which is one of the important signs of TCM case record maturity; B, TCM cases were recorded in monographs, collection, and research books. In particular, personal medical cases monographs flourished [31]; and C, TCM case records have become a specific medical literature style, which required not only to record personal clinical experience but also to comprehensively analyze the use of TCM theory.

As the rise of every TCM academic school is closely related to the background of the times, the emergence of the Warm Diseases School was a good example of a product of the call of the times of Ming Dynasty. At the end of the Ming Dynasty, major epidemics broke out in Hubei, Zhejiang, and Shandong provinces, resulting in countless deaths and fatalities. As a result, a physician of Wu Youke wrote the *Treatise on Pestilence* (*Wen Yi Lun*) and founded the Warm Diseases School, which was derived from the Typhoid School and He-Jian School and was known for its research and treatment of warm fever, which laid the foundation for the later development of epidemiology of exopathic diseases in China. The representative figures and works of the Warm Diseases School included Ye Tian-shi's *Treatise on Epidemic Febrile Diseases* (*Wen Re Lun*) and *Guide to Clinical Practice with Medical Records* (*Linzheng Zhinan Yi'an*); Wu Jutong's *Differentiation of Warm Febrile Diseases* (*Wen Bing Tiao Bian*); and Xue Shengbai's *Synopsis on Damp-Heat* (*Shi Re Bing Pian*). Han Mao (1441–1522 AD), an excellent doctor in Ming Dynasty, put forward the specific regulations on TCM case record in his monograph *Hanshi Yitong* (*Han's Medical Treatment*) [32], including inspection, auscultation, olfaction, inquiry, pulse-taking, palpation, diagnosis, and treatment. And he subdivided these regulations into 27 specific items [33, 34]. This was the first time for TCM doctors to put forward the standardized writing structure and elements of TCM case record.

Wu Kun (1551 AD–1620 AD), a successor of his medical family in Ming dynasty, has been engaged in medicine for nearly 60 years. He has written a total of 6 kinds of medical books, one of which was *Mai Yu* (*Essence of Pulse Theory*) [35]. He further supplemented and modified the writing structure and elements of TCM case record as “Seven Aspects and One Quotation.” Seven aspects of case record include the following: A, date of consultation, place, and patient's name; B, age, height, weight, face color, and voice; C, the main symptoms and signs, the onset time and the aggravated time of illness, and disease condition; D, the previous treatment history and clinical effects; E, nature of yin or yang, afraid of cold or heat, diet, sleep condition, and pulse condition; F, name of disease, name of TCM pattern, manifestation and root cause of disease, and chronic or acute; G, treatment method, diagnosis, prescriptions, the modification of prescription, the explanation of Chinese medicine and prescriptions, and contraindication. One quotation indicated that a doctor should sign his name and district at the end of a case record. However, he did not include any specific TCM case in his book.

Yu Jia-yan (1585 AD–1670 AD) was a famous doctor in the south of Yangtze River. His representative works, *Yuyi Cao* (*Experience of Chinese medicine*) [36], recorded more than 60 cases of miscellaneous diseases. Dr. Yu wrote a specialized chapter in the book about the standardization of writing content and format of TCM case record, including date of consultation, place, patient's name, age, height, weight, face color, voice, emotions, disease condition, etiology, the onset time and the aggravated time of disease, the main symptoms and signs, the present syndrome, the previous treatment history and clinical effects, afraid of cold or heat, diet, excretory functions, sleep condition, pulse condition, manifestation and root cause of disease, treatment method, diagnosis, prescriptions, composition of Chinese herbs, analysis of illness, analysis of treatment, and clinical efficacy. He not only ruled the specific contents of four diagnostic methods, but also advocated that we should pay attention to the external and internal environment for patients, at the same time record in detail the happening of the disease, the instant syndrome, and the therapeutic process. What's more, he emphasized that the doctor must write the evidence and principle of prescription clearly, as well as the prognostic situation. Thus, the comprehensive standardization of the items included in TCM case record played a crucial role in the training of clinical ability, the in-depth discussion of TCM theories, and the improvement of clinical level, and also had great practical significance to promotes the development of TCM case record.

Jiang Quan (1522–1566 AD) and his son collected all of the monographs on medical case records of their predecessors before Ming Dynasty and integrated them into *Mingyi Leian* (*Classified Medical Records of Celebrated Physicians*) [37], which was the first summary of TCM case records in Chinese history. The book consisted of 12 volumes, which were divided into 205 chapters with more than 3,300 TCM case records. It provided their pioneering experience for the compilation of TCM case records, which was important work linking the past with the future.

  Case 3: In August 1280 AD, a Judge Zhao of Zhengding County, Shijiazhuang City, Hebei Province, suffered a stroke. His signs and symptoms included loss of consciousness, paralysis on one side of his body, facial redness, deafness, nasal congestion, and slurred speech. Dr. Zhang felt his pulse, which showed stringy and rapid. From these manifestations, the diagnosis was a stroke that apoplexy infringed on the internal organs (zang-fu organs). He believed that a stroke in the five-zang organs would block the nine orifices, whereas a stroke at six-fu organs would stagnate the limbs. Therefore, he used Sanhua decoction as a purgative formula to eliminate blood stasis for regulating *qi* and nourishing limbs, and then used the *Zhibao Dan* (*zhibao mini-pills*) as inducing resuscitation formula to calming heart for tranquillization and resuscitation with aromatics in order to make *zang-qi* ascending and nine orifices resuscitated. After five days, the consciousness and speech turned clear. Dr. Zhang modified the prescription and asked the patient to use a rope to tie the affected low limb for walking aid exercise in order to promote the recovery of motor function. A few days later, the patient's muscle strength improved. During the stroke recovery period, Dr. Zhang performed acupuncture on the meridian points of the twelve regular meridians for dredging meridians and collaterals. The patient's motor function improved rapidly, and he was able to walk several hundred steps without support. It was obvious that the patient was recovering well. Dr. Zhang told him to maintain a good mood and a regular diet every day. At the follow-up visit one year later, the condition and quality of life had basically improved (From *Classified Case Records of Celebrated Physicians*).
  Comments: Zhang Yuan-su was a famous doctor in the Jin Dynasty (1115–1234 AD) who specialized in treating stroke. This case is written in a more complete format and content, as it contains symptoms, syndromes, basic information, diagnosis, treatment, and follow-up. It has a time line depicting important dates and times. It also reports the therapeutic method of each treatment and the doctor's own views of the disease. As for the Sanhua decoction used by Dr. Zhang, our modern pharmacological study found that it can alleviate neurological deficits and exert neuroprotection against focal cerebral I/R injury [38].

Wei Zhi-xiu (1722–1772 AD), an eminent doctor, compiled a monumental work of *Xu “Mingyi Leian”* (*Supplement to “Classified Case Records of Celebrated Physicians”*) [39], which was considered the great work and second summary of TCM case records. It initially finished 60 volumes in 1770 AD. Unfortunately, he left his incomplete works since he died suddenly. Wang Shi-xiong, a famous peer, continuously completed the Wei's work and reclassified the book into 36 volumes, 350 chapters. This book collected about 5, 000 TCM case records from 308 famous physicians before the early Qing Dynasty. It takes the case records under the name of disease, and thus, one disease has several case records. The readers could compare mutually and inspire among them. In addition, Dr. Wei's detailed description of the causes of most of the patients includes their previous history, hobbies, dietary habits, body constitution, mental state, and family background. It not only provided comprehensive data for his successors to analyze TCM but also lay a foundation for promoting the development of case records. Until now, it was still the largest extant writings of TCM case records, which was of high value in both literature and academic.

Li Shi-zhen (1518–1593 AD), one of the greatest physicians, pharmacologists, and naturalists, compiled the outstanding medicine monograph *Bencao Gangmu* (*Compendium of Materia Medica*) [40], which is a work on an encyclopedic scale. He attached the relevant TCM case records below each herbal medicine in order to provide corroborative evidence of the efficacy of the medicinal product.

Ye Tian-shi (1667–1746 AD) was considered an eminent doctor in infectious disease in TCM. His followers collected his medical cases in his later years and compiled them into a book named *Linzheng Zhinan Yi-an* (*Medical Records as a Guide to Clinical Work*) [41]. Since its publication in 1764 AD, the book has become one of the most researched, the most numerous editions and the most reprinted personal medical monographs. There are 10 volumes in total, including medical, surgical, gynecological, paediatric, and androgynous diseases and syndromes. All the TCM case records reflected Dr. Ye's academic inheritance and innovation from the predecessors. TCM cases were recorded in chronological order of the symptoms, pathogenesis, dialectical diagnosis, and prescriptions according to the process of before and after treating. Its content was relatively comprehensive and has rich and profound meaning, which still plays an important role in the enlightenment of later generations to learn about TCM case records [42].

In the late Qing Dynasty, Western medicine gradually developed in China, and progressive physicians with a reform spirit in the TCM team realized that both Chinese and Western medicine had their own strengths and tried to converge the two academic disciplines. They put forward a series of views on the convergence of Chinese and Western medicine from theory to clinical practice and gradually formed a convergence trend and the School of Fusion of Chinese and Western Medicine. The School of Fusion of Chinese and Western Medicine was represented by Tang Zonghai, Zhu Peiwen, and Zhang Xichuan, whose representative works include *Records of Chinese medicine and Western Medicine* (*Yixue Zhongzhong Canxi Lu*) and *Five kinds of Chinese and Western Medical Books*. Zhang Xichun, was eclectic, absorbing new knowledge on the basis of traditional medicine and publishing his academic views and his clinical experience in the press continuously, which was widely praised by the medical community and had a great impact on the Chinese and Western medicine, making Zhang Xichun one of the most representative figures of the School of Fusion of Chinese and Western Medicine. This school opened up a new trend of convergence between Chinese and Western medicine and opened up the way for the modern combination of Chinese and Western medicine.

## 4. Modern Period

The modern period refers to the period from the Republic of China (1912–1949) to the present. Western medical knowledge has been permanently introduced into China [43]. Especially after the Opium War, a large number of missionaries were crowded into China [44], leading to unprecedented challenges in a variety of fields. As a result, many TCM practitioners began to integrate Western medicine into their practice and established medical journals for TCM.

After the establishment of the Republic of China in 1912, the whole governmental system was modeled on a western pattern, including the medical system. Thus, the development of TCM has not last long before because the Republic of China government changed the policy to restrict the medical activities of TCM and even call for abolishing TCM in 1929. TCM case records have also undergone a hard and tortuous course of development. TCM doctors began to vigorously establish medical journals, which can help to exchange medical experience and skill quickly. According to statistics, the number of TCM periodicals created during the Republic of China was at least 378. The spread media of TCM case records have partly turned from the books to journals, which leads to a transformation from case records to case reports in terms of writing format. For example, He Lian-chen (1861–1929), a prominent physician in the modern TCM history, recorded the case of scarlet fever using modern diagnosis and examination tools such as laryngoscope and round mouth plier to help diagnosis in his book *Chongding “Guang Wenre Lun”* (*The Revise of “Treatise on Epidemic Febrile Diseases”*) [45]. He also founded the earliest TCM journal *Acta Medica Shaoxing* in June 1908 [46] and set up a case column that classified the specific items as follows: A, patient information: name, gender, age, and address; B, history of present illness: etiology, onset time of disease, and symptoms. He emphasized the patient's name and clinical effect should be recorded clearly because it is good for later generations to verify the authenticity and accuracy so as to the value of modern medical record. He also pointed out that it is necessary to record the authors' institutions or societies, or any other relevant background information in published clinical case report [47]. He proposed eight requirements for the purpose of regulating writing format of TCM case to a unified style, which included patient, name of disease and name of TCM pattern, etiology, TCM pattern, diagnosis, treatment principle, prescriptions, and treatment outcomes. Based on the eight requirements, he further selected 371 case records from national prominent veteran TCM doctors and compiled them into a book *Quanguo Mingyi Yanan Leibian* (*Compilation of TCM Case Records from National Prominent Veteran TCM Doctors*) [48] in 1922.

Case column of a journal was gradually germinated and developed. The columns of TCM case report were mainly divided into four categories during the period of the Republic of China: (1) medical cases of famous doctors—the column of medical cases of famous doctors at that time was fresh records of clinical diagnosis and treatment in terms of content. There are also some columns, due to the long-term publication of a certain doctor's experiences, gradually developed into a column to record his or her individual clinical cases, such as *Ding Ganren Yan-an* (*Experienced Treatment Cases of Dr*. *Ding Ganren*) [49]; (2) ancient medical cases—the column of ancient medical cases reviewed the TCM case records of influential predecessors and put forward their own opinions about the cases, so as to strengthen the understanding and further study of TCM cases, such as *Gujin Yi-an Pingyi* (*Review on Ancient and Modern TCM Case Records*) [50] (written by Dr. Zhang Shanlei); (3) medical cases on quitting opium—the column of medical cases on quitting opium was emerged because Western countries began and smuggle opium into China, such as *Cao Yingfu Yi-an* (*Medical Case Records of Cao Yingfu*) [51]; and (4) medical cases about infectious diseases—due to the changeable climate and the poor sanitary conditions, many TCM journals opened up columns that actively participated in the prevention and treatment of epidemic diseases such as cholera, measles, and encephalitis, and the publications were more than 1,000 articles [52]. Thus, the publication of medical cases on opium cessation and infectious diseases was in line with the trend of that era.

As case records in medical books are still important as before, we summarized the content of the format standard of TCM case records from the 5 monographs, which is related to the format standard of medical cases, and then extracted the structure and elements of TCM case records (Table 4). In addition, according to the writing habit of modern medical cases, we combined the structures and elements obtained by the same category and counted the frequency of structures and elements according to the corresponding attribution principle (Table 5). The results showed that the ancient TCM case records not only included some items of the modern medical records such as the basic information, chief complaint, disease history, diagnosis, and treatment, but also contained the contents such as inspection, listening and smelling, inquiring, and pulse-taking, which can reflect the characteristics of TCM. The TCM case records especially emphasized the content of medical case analysis, which highlighted the essential attribute that case records are the carrier of clinical experience.

Since the People's Republic of China had established on October 1, 1949, the country and government have attached great importance to TCM. The Chairman Mao once said: “Traditional Chinese Medicine is a great treasure-house and should be explored and improved diligently.” At present, the economic strength in China has enhanced greatly, and modern science and technology has developed rapidly, providing favorable social, economic, and technological conditions for the development of TCM [53]. The development of TCM case record/report is presented a situation of a hundred flowers bloom as follows: A, the establishment and development of the discipline of TCM medical records; B, the writing format of TCM medical records is standardized; C, lots of books concentrated on recording and studying TCM case records, especially from prestigious veteran doctors; D, a surge of TCM case reports published in journals; and E, establishment of TCM case database and application platform integrating computer program with artificial intelligence.

With the development of TCM case records, the discipline of TCM medical records has been set up [54, 55] for the purpose of analyzing, extracting, and summarizing TCM case record and then deeply studying the law of diagnosis and treatment, the clinical thinking characteristics, academic thought, and corresponding research methods of TCM [56]. As a new branch of TCM disciplines, the discipline of TCM medical records has been developing rapidly. The increasing number of medical schools has compiled the corresponding textbooks for undergraduate and graduate students, such as “*Introduction to Medical Cases of Modern Prominent Veteran TCM Doctors*” [57]. Thus, the discipline of TCM medical records shows a significant role in the education field. On the one hand, case studies have the potential to be highly read and to have a significant impact on subsequent case research [58]. It provides a wide range of subjects, ideas, and methods for graduate students to carry out clinical, experimental, and history literature case research, and also plays a significant role in cultivating graduate students' clinical practice skill and scientific methods [59]. TCM case record can also help students or practitioners to learn relevant information and technology, which may strengthen clinical thinking ability and sense of responsibility [60]. It not only gives readers a chance to confront novel clinical scenarios and reflect upon their own practice [61], but also trains authors to think and write clearly and critically case records [62].

The writing format of TCM medical records become gradually standardized. In 1983, the Department of TCM in the Ministry of Public Health formulated and issued “*The writing format and requirements of TCM medical records (trial)*.” In 1991, the *National Administration of Traditional Chinese Medicine* formally formulated the writing regulations of TCM medical records and published the book “*Standards of Writing TCM medical records*” [63]. The regulations include five aspects: A, general rules of writing TCM medical records; B, unified name of TCM medical records; C, sort order and explanation of subjects of TCM medical records; D, writing format of TCM medical records; and E, key points of writing medical records about various TCM departments and set examples of them. In 2002, the *National Administration of Traditional Chinese Medicine* set up “*The general standards of writing medical records of TCM and integrated Chinese and Western medicine (trial)*” [64]. In 2010, the *National Administration of Traditional Chinese Medicine* promulgated “*The general standards of writing TCM medical records*” [65]. These regulations issued by the national administration promoted the development of medical records and gradually transformed the traditional writing format of TCM case records into “modern medical records.” These regulations require a complete set of items, including TCM characteristic diagnosis and treatment information such as inspection, auscultation, smelling, inquiry, and pulse-taking.

Lots of books concentrated on recording and studying TCM case records. One of the representative literature studies is “*ErXu ‘Mingyi Leian”'* (*The Second Supplement of ‘Classified Case Records of Celebrated Physicians'*)” [66], compiled by Lu et al. in 1996. It was regarded as the second supplement of “*Classified Case Records of Celebrated Physicians*” and the third summary of TCM case records. This book added the medical cases of famous doctors from the Qing Dynasty to the early years of People's Republic of China, with a total of about 15,000 medical cases and more than 200 monographs of TCM case records. In 1990, the *Ministry of Personnel*, *Ministry of Health, and National Administration of Traditional Chinese Medicine* jointly issued the decision on taking emergency measures to inherit the academic experience of prestigious veteran TCM doctors [67]. Due to the urgency to rescue their academic thoughts and clinical experiences, a large amount of case records by prestigious veteran TCM doctors have been compiled and published across China. The first kind is a compilation of medical cases of one famous TCM doctor such as *Medical Records of Pu Fuzhou*, which was first published in 1972 and was regarded as a classic work of TCM medical cases after the founding of the People's Republic of China [68]. The second kind is the book on collecting, organizing, and researching various medical cases of modern TCM doctors such as *The Essence of Medical Records of prestigious veteran TCM doctors*, which was compiled in 1990 and a selection of 146 famous TCM doctors with 1850 cases [69]. It reflected the general scenery of contemporary TCM clinic and played an important role in summarizing and inheriting the clinical experience of old TCM practitioners [70]. The third kind is medical case monographs on specific departments, which are on the rise. *Review and Analysis of Medical Case Reports in Internal Diseases by Famous Doctors* [71], edited by Li et al. [72], took the classification of internal diseases as the outline and the classification of doctors and TCM diseases and syndromes as the order. It compiled the TCM case reports of nearly 100 influential doctors, which promotes the development of TCM case reports of specialized departments. All in all, summarizing, inheriting, and studying the experience and academic thoughts of prestigious veteran TCM doctors can not only enrich the theoretical system of TCM, but also promote the academic progress of TCM [72].

**Figure 2:**
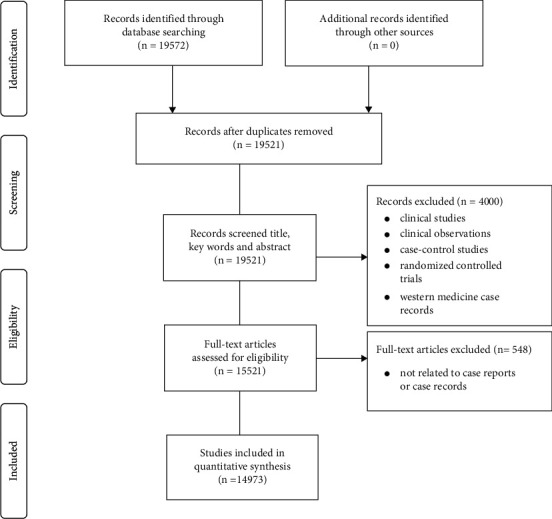
Flow diagram of the included studies.

**Figure 3:**
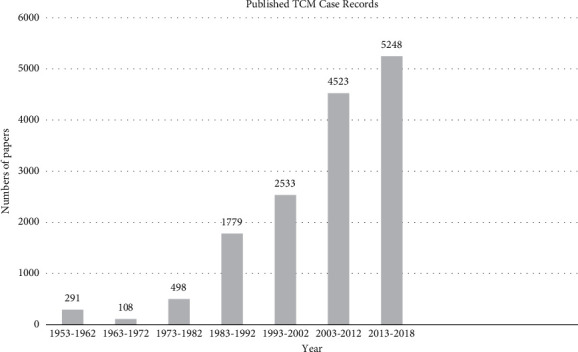
The characteristic of publishing trend of TCM case records (1953–2018).

A number of medical journals and columns have set up that focus on case reports. The first international, PubMed-listed medical journal called “*Journal of Medical Case Reports*” was established in 2007 [73]. Up to 2013, thousands of medical journals listed in PubMed have published more than 1.6 million medical cases [74]. At mid-2015, more than 160 journals were set up specifically for reporting case reports [75]. We did electronic searches in the database of Chinese National Knowledge Infrastructure from the inception to December 31, 2018, with the search terms of “subject” containing “case report^*∗*^” OR “case record^*∗*^” OR “case series” OR “medical case^*∗*^” OR “medical record^*∗*^,” excluding “case control” AND “case observation” AND “clinical study” AND “clinical observation” AND “randomized controlled trials,” and the literature source set from “Traditional Chinese medicine.” A total of 19572 studies were identified, of which 51 studies were excluded because of duplicates. After screening the title and abstract of studies, 4000 studies were excluded because they are observations of clinical efficacy, minutes of conferences, or western medical case records. Five hundred and forty-eight studies were excluded because they are not related to case reports or case records after browsing full text. Finally, there were 14973 TCM case records or case reports left. The process of screening is presented in a flow diagram (Figure 2). The bar chart (Figure 3) shows that the general trend of publishing TCM case reports in journals was growing. It has a well beginning because TCM was used as a symbol for China and considered a national treasure in the years between 1953 and 1959 under the chairman Mao Zedong' government. However, when the Cultural Revolution was launched, traditional culture, thinking, and perspectives were crushed. TCM and TCM practitioners also suffered greatly under this movement. As a result, the number of published TCM case reports was decreased. After the Cultural Revolution was officially ended in 1976, TCM and Western physicians slowly re-emerged into China's society and TCM began to revive, which led to the ascending number of published cases. Since entering the 21st century, TCM has developed rapidly with its strong vitality. Between 2003 and 2012, the number of TCM case reports was nearly twice as much as that of the past decade, and the number of TCM cases reported in the recent five years exceeded the total number of cases reported in the previous ten years. Thus, the situation of publication of TCM case reports has increased rapidly in the past decades, indicating a great interest in the area.

The research methods of TCM case records have been advanced because of the application of computer program and artificial intelligence. With the combination of mathematical and statistical methods, many databases established specifically for TCM case records [76]. For example, Chinese Medical Case Database [77], Statistics and Analysis System for Medical Case Records in Ancient and Modern Times [78], and Database of Ancient TCM Medical Cases [79] resulted in much improvement in the efficiency of consulting case records literature. Computer software of an expert system [67] is an application platform for the purpose of exploring the medical thought and clinical experiences contained in TCM case records, so as to find the laws of clinical diagnosis and treatment of famous TCM practitioners, which can quickly transform doctors' experience into theory and guide clinical practice. According to artificial intelligence, an evidence base of TCM case reported system has been initiated to use in the Donzimeng Hospital of Beijing University of Chinese Medicine. All these high-tech tools serve as a breakthrough in the case record research method [80] help to improve the reporting quality and contribute to inheriting and developing TCM [81].

As for TCM academic school, since the Jin-Yuan Dynasty, they have been flourished and different distinctive schools of Chinese medicine have emerged so far. For example, according to the geographical division, there is the Yi-Shui School in the north, the Meng-He school in the south, and other schools such as the Qian-Tang school, Xin-An school, and Long-Sha school, which have flourished and led the way. The Hai School of Chinese Medicine has a history of several hundred years and has been divided into many specific academic schools according to different disciplines.

On July 30, 2013, the kick-off meeting of the national TCM academic school inheritance studio construction project, sponsored by the National Administration of Traditional Chinese Medicine and hosted by the Traditional Chinese Medicine Bureau Of Guangdong Province and Guangzhou University of Traditional Chinese Medicine, was held in Guangdong. The smooth and successful convening of this project kick-off meeting not only beat the gong for the full-scale work of the first batch of 64 TCM academic schools nationwide, but also marked the establishment of the TCM academic schools of TCM Inheritance Promotion Base Office, which is responsible for the daily business guidance and provision of services for the academic schools of TCM inheritance studios. This is conducive to promoting academic exchanges and resource integration among academic schools, realizing the intensification, scale, and specialization of academic school inheritance promotion, promoting the common development and sharing of results among schools, building a service platform for each school studio, promoting exchanges among academic schools, accelerating research, and realizing the transformation of results. With the change of times, there are many problems and challenges in the development of TCM academic schools. For example, there is a single mode and method of training talents of schools, the lack of communication among schools conservatively, the constraints of standardization and standardization of TCM treatment on the development of school inheritance, and the imperfect evaluation and use mechanism of school talent training. The characteristics of TCM clinical treatment are reflected in personalized treatment plans, reliance on doctors' personal experience, and the existence of multiple choices of TCM theories to guide clinical practice, rather than a single standard. Therefore, while implementing the standardization and standardization of TCM clinical and case writing, it is necessary to fully respect the law of TCM's own development, not only to meet the requirements of the accepted TCM theories and technical specifications, but also to reserve enough space for the development of unique academic ideas, clinical skills, and treatment characteristics, so that the TCM academic school can not only retain their own characteristics in terms of content, but also keep pace with the times, constantly accommodating and absorbing new elements, and promote their format tends to be standardized and standardized.

### 4.1. The Standardization of TCM Case Reports

The reporting quality of cases varies considerably in medical journals [82]. One study [83] had evaluated 1,316 case reports, which were published in four emergency medicine journals. The result showed that most case reports had fully reported patient information, types of interventions, and outcomes, whereas 50% case reports had not detailed the specific interventions and 67% case reports had not mentioned adverse events. Thus, in recent years, many experts in journals and organizations have developed reporting guidelines to increase the reporting quality of case report in both conventional western medicine and complementary and alternative medicine (CAM). In 2013, a consensus-based clinical case reporting guideline [84] was proposed through a consensus process in order to improve the completeness and transparency of published case reports. The CAse REport (CARE) guideline was simultaneously published in 7 journals. Up to 2017, this guideline has been translated into 10 languages and endorsed by a number of medical journals [85].

Similarly, the necessary elements of a CAM or TCM case had been continuously proposed for the purpose of providing guidance for publication in a standardized format in a peer-reviewed journal (Table 6). In 2004, the editor-in-chief Adrian White in the journal of “*Acupuncture in Medicine*,” had written two articles [86] entitled “*Writing case reports – author guidelines for Acupuncture in Medicine*” in order to help authors to write thorough but succinct case reports in a structured manner. *Guidelines for case reports of adverse events related to acupuncture* had been published in the same year and same journal [87]. In 2008, based on the international requirements for case report writing, Yang et al. [81] integrated with the characteristics of the TCM theory and summarized the key components of TCM case reports, which can be used as a reference for later TCM practitioners to write case reports, and for qualitative quality evaluation of published case reports. In 2009, Wang et al. [88] propounded the recommendations on the publication and process norms of clinical case and determined the writing requirement and traceability from the aspects of brief abstract, background, general information, chief complaint, disease history, diagnosis, treatment, discussion, expert estimate, and reference.

van Haselen [89] presented a conceptual framework in 2015 for developing clinical case reporting guidelines for CAM treatments to integrate general guideline with specific quality items of CAM therapy. This framework has been practically implemented the development of a reporting guideline for case report in homoeopathy. It will be more clarity and transparency in reporting CAM cases because the specific quality items of CAM treatment are determined by the specific characteristics of the clinical case report and the corresponding specific objectives of CAM discipline. It will also greatly contribute to CAM research and education, as well as to improved patient care.

In 2016, a working group of case report in Chinese medicine (CARC) established systematic recommendations by reviewing the general reporting quality of case reports [9]. The CARC recommendations comprised a 16-item checklist, including title, abstract, keywords, English summary, introduction, patient information, clinical findings, diagnosis, treatment, outcome assessment, follow-up, advices and precautions, discussion/comment, acknowledgment, references, and figures/tables. They can not only expound the specialties of TCM, but also can apply to the common projects in modern health research. Thus, they have an important effect on promoting the development of TCM as it retained the principles of scientific, diversity, and practicability and satisfied the needs of standardization.

In the same year, Adams [90] developed an introductory guide for practitioners to prepare case reports on herbal medicine. The checklists are as follows: title, author(s), addresses and any affiliation, abstract, introduction, case report, literature search, discussion, conclusion, acknowledgments, references and tables, illustrations, photographs, and figures. He points out the important issue that it is not wise to give a biomedical interpretation for the response to your herbal treatment without supporting references. The solution is that we can humbly point out that the activity of herbal medicine can only be explained in general terms by previous clinical observations due to the lack of knowledge and inadequacy understanding of many biomedical mechanisms. By following the recommendations and checklists of these guidelines, the reporting quality of TCM case reports would be greatly improved.

## 5. Conclusions

TCM case records have a history of several thousands of years and have been continuously inherited and developed. This study analyzed and illustrated the characteristics of TCM case records of different dynasties in terms of writing content and format. In modern times, with the strengthening of China's economic strength and an increase of policy support for TCM, TCM case records have also gained a rapid development in the following aspects: the state attaches great importance to the transmission of academic thoughts of prestigious veteran TCM practitioners; published a large number of medical case monographs; the emergence of a large number of specialized clinical cases both in books and journals; attached importance to standard writing of medical cases; and the application of technological innovation research methods such as mathematical statistics, database, and artificial intelligence. Despite its ancient origins, the discipline of TCM case records is a relatively young discipline. TCM case records remain far-reaching significance for the inheritance and development of TCM theory and clinical experience. From the wisdom of history, its positive impact has just been revalued to be validated and it will continue to develop.

## Figures and Tables

**Table 1:** Summary of main contributions of TCM case records in the early period.

Representative books	Author	Time	Contributions to TCM case records
*Rites of Zhou*	Zhou Gong-dan	—	It recorded the earliest organized medical system in the Eastern Zhou dynasty in which the imperial officials received various medical training. TCM doctors were classified into four categories: *shiyi* (dietitian), *jiyi* (physician), *yangyi* (surgeon), and *shouyi* (veterinarian). The doctors' salary was connected with their medical cases recorded by the chief doctor. The death was needed to record too.

*The Spring and Autumn of Lv Bu-wei*	Lv Bu-wei	239 BC	It is an encyclopedic Chinese classic document, which had recorded the budding of TCM case record.

*The Historical Records*	Sima Qian	206 BC-24 AD	There were 25 TCM cases recorded in the biographical section of *The Historical Records*, named *Biography of Bian Que and Cang Gong*, which was probably the earliest extant and relatively complete TCM case records in Chinese historical literature. Cang Gong was the first to record clinical cases through personal observations with the motivation to evaluate the percentage of successes and failures and find a guide for future predictions. The 25 cases of him reflected the change of thinking from passive assessment to active case compilation in the Western Zhou dynasty.

—: not clear; TCM: traditional Chinese medicine.

**Table 2:** Summary of main contributions of TCM case records in the middle period.

Representative books	Author	Time	Contribution to the TCM case records
*Ninety syndromes of typhoid diseases*	Xu Shu-wei	1133 AD	It was the first existing TCM case records monograph, and the earliest collection of typhoid case records. The book described ninety cases of typhoid.

*Prescriptions of formularies for universal relief*	Xu Shu-wei	1143–1154 AD	It was the representative formularies that compiled TCM case records in the form of an appendix. He was the pioneer to record clinical cases behind his prescriptions, which expounded his TCM theory and had an impact on the format of TCM case record.

*The appropriate way of recognizing and treating infant maladies*	Qian Yi's disciple	1119 AD	It was the earliest extant, well-preserved, and complete monograph of paediatric case records in China written by Qian Yi's disciple. In the view of Dr. Qian's cases, we deduced that he was the pioneer to differentiate the symptoms of diseases.

*Han's medical treatment*	Han Mao	1522 AD	It was firstly to put forward the specific regulations on TCM case record, including inspection, auscultation, olfaction, inquiry, pulse-taking, palpation, diagnosis, and treatment. These regulations were subdivided into 27 specific items. This was the first time for TCM doctors to put forward the standardized writing structure and elements of TCM case record.

*Essence of pulse theory*	Wu Kun	1584 AD	It further supplemented and modified the writing structure and elements of TCM case record as “seven aspects and one quotation.”

*Experience of Chinese medicine*	Yu Jia-yan	1643 AD	It contained a specialized chapter about the standardization of writing content and format of TCM case record. It completed the standardization of the items in TCM case record, which played a crucial role in the training of clinical ability, the in-depth discussion of TCM theories, and the improvement of clinical level and also had great practical significance to promotes the development of TCM case record.

*Classified medical records of celebrated physicians*	Jiang Quan and his son	1549 AD	It collected all of the monographs on medical case records of their predecessors before Ming dynasty, which was the first summary of TCM case records in Chinese history. It provided their pioneering experience for the compilation of TCM case records, which was important work linking the past with the future.

*Supplement to classified case records of celebrated physicians*	Wei Zhi-xiu and Wang Shi-xiong	1770 AD	It was considered the great work and second summary of TCM case records. It not only provided comprehensive data for his successors to analyze TCM but also lay a foundation for promoting the development of case records. Until now, it was still the largest extant writings of TCM case records, which was of high value in both literature and academic.

*Compendium of Materia Medica*	Li Shi-zhen	1578 AD	It was the first time to provide corroborative evidence of the efficacy of the medicinal product through attaching the relevant TCM case records below each herbal medicine.

*Medical Records as a Guide to Clinical Work*	Ye Tian-shi	1764 AD	It has become the most researched, the most numerous edition, and the most reprinted personal medical monograph. Its record content was relatively comprehensive and has rich and profound meaning, which still plays an important role in the enlightenment of later generations to learn about TCM case records.

TCM: traditional Chinese medicine.

**Table 3:** Summary of main characteristics of TCM academic schools in Jin and Yuan dynasties.

Academic schools	Representative books	Author	Time	Main theory	Similarities	Differences
He-Jian school (fire-heat school)	*Plain Questions on Concept of Original Disease Type*	Liu Wan-su	1110 AD–1200 AD	According to the “five evolution phases and six climatic factors” principle, he advocated the theory that six climatic factors can transform fire.	The theory was referred to the classical Chinese medical work of Huangdi's internal classic.	1. He used the “nineteen articles of pathogenesis” of plain questions as the theoretical basis, systematically classified diseases according to the five evolution phases and six climatic factors (five Yun and six Qi). 2. He also proposed the principles of cooling the exterior of the body and dipping heat to nourish yin as the treatment of heat-related diseases, which laid the foundation for the formation of the later warming diseases school.

Eliminating pathogenic factor school	*Confucians' Duties to parents*	Zhang Cong-zheng	1156 AD–1228 AD	He emphasized the academic idea of “disease is born from pathogenic factors.”	He was a follower of Liu Wansu, who also influenced the warming diseases school.	He developed the method of eliminating pathogenic factors by sweating, vomiting, and coming down methods.

Tonifying spleen school	*Treatise of Spleen and Stomach*	Li Dongyuan	1180 AD–1251 AD	He believed that the internal injury to the spleen and stomach or inadequate spleen and stomach can lead to all kinds of diseases.	His theory was based on the theory of Huangdi's internal classic	He thought that the four seasons are all based on nourishing stomach qi, so his treatment emphasized on regulating the spleen and stomach and raising the middle qi, and he made new formulas such as Buzhong Yiqi decoction.

Nourishing yin school	*Danxi's Mastery of Medicine*	ZhuDan-xi	1281 AD–1358 AD	He created the theory that “Yang is always in excess and Yin is always in deficiency,” advocating the nourishment of Yin.	He was the third-generation disciple (direct student) of Liu Wansu and had studied the doctrines of the above three schools.	1. He advocated the doctrine of “nourishing yin” with Yin deficiency and Yang hyperactivity as the core, and created the famous formula, Yue Ju Pill (Yue Ju Wan). 2. *Danxi's Mastery of Medicine* was compiled by his disciples and focuses on miscellaneous internal diseases and the Danxi doctrine, discussing in detail the disease name, cause, syndrome, identification, symptoms, and treatment prescriptions. 3. The nourishing Yin school had an important influence on the formation and development of the later Xin'an school of medicine.

**Table 4:** The main structure and elements of the format standard of TCM case records from the 5 monographs.

Representative books	Structure and elements
*Biography of Bian Que and Cang Gong*	Patient' name, home address, profession, name of disease and name of TCM pattern, main complaint, pulse condition, etiology, pathogenesis, syndrome differentiation, diagnosis, treatment, and prognosis

*Han's Medical Treatment*	Date of consultation, place, height, weight, face color, voice, disease condition, disease location, etiology, the onset time and the aggravated time of illness, afraid of cold or heat, diet, the previous treatment history, pulse condition, manifestation and root cause of disease, diagnosis, severity of illness, treatment method, prescriptions, medication methods.

*Essence of Pulse Theory*	Date of consultation, place, patient's name, age, height, weight, face color, voice, the main symptoms and signs, the onset time and the aggravated time of illness, disease condition, the previous treatment history and clinical effects, nature of yin or yang, afraid of cold or heat, diet, sleep condition, pulse condition, name of disease, name of TCM pattern, manifestation and root cause of disease, chronic or acute, treatment method, diagnosis, prescriptions, the modification of prescription, the explanation of Chinese medicine and prescriptions, contraindication, the doctor's signature.

*Experience of Chinese Medicine*	Date of consultation, place, patient's name, age, height, weight, face color, voice, emotions, disease condition, etiology, the onset time and the aggravated time of illness, the main symptoms and signs, the present symptoms, the previous treatment history and clinical effects, afraid of cold or heat, diet, excretory functions, sleep condition, pulse condition, manifestation and root cause of disease, treatment method, diagnosis, prescriptions, composition of Chinese herbs, analysis of illness, analysis of treatment, clinical effect.

*Eight Requirements to TCM Case Record and Case Report by He Lian-chen*	Patient, name of disease and name of TCM pattern, etiology, TCM pattern, diagnosis, treatment principle, prescriptions, treatment outcomes.

TCM: traditional Chinese medicine.

**Table 5:** The frequency of structures and elements according to the corresponding attribution principle.

Structure (frequency)	Corresponding attribute element (frequency)	
Patient information (5)	Date of consultation (3), place (3), patient's name (3), age (2), home address (1), profession (1)	
Past medical history (3)	The previous treatment history (2) and the previous clinical effects (3)	
Diagnosis (5)	Inspection (4)	Height (3), weight (3), face color (4)
	Auscultation (3)	Voice (3)
	Inquiry (5)	The main symptoms and signs (2), the onset time of illness (2), the aggravated time of illness (3), afraid of cold or heat (3), diet (3), sleep condition (2), excretory functions (1), emotions (1)
	Pulse-taking (5)	Pulse condition (4)
	Other diagnoses (2)	Name of disease (3), Name of TCM pattern (3)
Treatment (5)	Treatment method (4), treatment principle (1), prescriptions (4), medication methods (1), composition of Chinese herbs (1)	
Analysis of disease (2)	Etiology (4), pathogenesis (manifestation and root cause of disease) (3), nature of yin or yang (1), disease condition (3), disease location (1), severity of illness (1), chronic or acute (1)	
Analysis of treatment (2)	The modification of prescription (1), the explanation of Chinese medicine and prescriptions (1), contraindication (1)	
Outcome assessment (3)	Clinical effect (3)	
Prognosis (1)	Prognosis (1)	
Acknowledgment (1)	The doctor's signature (1)	

TCM: traditional Chinese medicine.

**Table 6:** The characteristics of guidelines of TCM case reports.

Title	Theme	Author	Time	Journal	Characteristics
Writing case reports—author guidelines for acupuncture in medicine	Acupuncture	Adrian White	2004	Acupuncture in Medicine	It was a developing guideline for helping authors to write thorough but succinct case reports in a structured manner. The format of acupuncture case report in this guideline includes an abstract, description of the case, literature search, discussion, and summary or conclusions. A patient consent is required before publication.

Towards improving the reporting quality of clinical case reports in complementary medicine: Assessing and illustrating the need for guideline development	Conventional and complementary medicine	R. A. van Haselen	2015	Complementary therapies in medicine	It was presented as a conceptual framework for developing clinical case reporting guidelines for CAM treatments to integrate general guideline with specific quality items of CAM therapy, which practically implemented the development of a reporting guideline for case report in homoeopathy. It will be more clarity in reporting CAM cases because the specific quality items of CAM treatment are determined by the specific characteristics of the clinical case report and the corresponding specific objectives of CAM discipline.

Consensus-based recommendations for case report in Chinese medicine (CARC)	Chinese medicine	FU Shufei	2016	Chinese Journal of integrative medicine	The CARC group established systematic recommendations by reviewing the general reporting quality of case reports. They have an important effect on promoting the development of TCM as it retained the principles of scientific, diversity, and practicability and satisfied the needs of standardization.

Writing a case report an introductory guide for practitioners of herbal medicine	Herbal medicine	Richard Adams	2016	Journal of herbal medicine	It was developed as an introductory guide for practitioners to write a case report about herbal medicine. It suggested a trick that herbal medicine' activity can only be explained in general terms with references of previous clinical observations partly because of inadequacy understanding of many biomedical mechanisms.

TCM: traditional Chinese medicine; CAM: complementary and alternative medicine.

## Data Availability

No data were used to support this study.
